# Unveiling the biochemical and haematological profile of blue shark (*Prionace glauca*) in the Mediterranean after bycatch

**DOI:** 10.1093/conphys/coaf067

**Published:** 2025-09-20

**Authors:** Lola Toomey, Andrea Bellodi, Massimiliano Bottaro, Antonella Consiglio, Eleonora Fiocchi, Margherita Soncin, Valentina Bertazzo, Maria Cristina Follesa, Amedeo Manfrin, Simone Niedermüller, Giulia Prato, Pierluigi Carbonara

**Affiliations:** COISPA, Fondazione COISPA ETS, via dei Trulli 18/20, 70126, Bari, Italy; Department of Life and Environmental Sciences, University of Cagliari, via T. Fiorelli 1, 09126, Cagliari, Italy; Department of Integrated Marine Ecology, Calabria Marine Center, Stazione Zoologica Anton Dohrn (SZN), C.da Torre Spaccata, Località Torre Spaccata, 87071 Amendolara, Italy; Department of Integrative Ecology, Genoa Marine Centre, Stazione Zoologica Anton Dohrn, Villa del Principe, Piazza del Principe 4, 16126, Genoa, Italy; COISPA, Fondazione COISPA ETS, via dei Trulli 18/20, 70126, Bari, Italy; Reference Laboratory for Fish, Mollusc and Crustacean Disease, Istituto Zooprofilattico Sperimentale delle Venezie (IZSVe), Viale dell’Università, 10, 35020, Legnaro, Italy; SCS3-Laboratory Medicine, Istituto Zooprofilattico Sperimentale delle Venezie (IZSVe), Viale dell’Università, 10, 35020, Legnaro, Italy; SCS3-Laboratory Medicine, Istituto Zooprofilattico Sperimentale delle Venezie (IZSVe), Viale dell’Università, 10, 35020, Legnaro, Italy; Department of Life and Environmental Sciences, University of Cagliari, via T. Fiorelli 1, 09126, Cagliari, Italy; Reference Laboratory for Fish, Mollusc and Crustacean Disease, Istituto Zooprofilattico Sperimentale delle Venezie (IZSVe), Viale dell’Università, 10, 35020, Legnaro, Italy; WWF-Mediterranean, WWF, via Po 25/c, 00198, Rome, Italy; WWF-Italy, WWF, via Po 25/c, 00198, Rome, Italy; COISPA, Fondazione COISPA ETS, via dei Trulli 18/20, 70126, Bari, Italy

**Keywords:** Bycatch, immunity, Mediterranean sea, osmoregulation, *Prionace glauca*, sex

## Abstract

The blue shark (*Prionace glauca*) is the most frequently by-caught species in longline fisheries targeting swordfish in the Mediterranean Sea. The IUCN classifies the Mediterranean blue shark population as critically endangered, but no information on haematological or biochemical parameters is available for this population. Based on a multi-year dataset of 63 blue sharks (*Prionace glauca*) and 18 physiological parameters, this study provides the first detailed insights into the variability of physiological indicators following bycatch in the Mediterranean Sea. We also examined differences across three post-capture condition groups and assessed the potential influence of sex and life stage (juvenile vs. adult) on physiological variability. While no significant differences emerged between sexes or life stages, clear distinctions were observed between condition groups, particularly when compared to moribund or dead individuals (condition group 3). These sharks showed signs of enhanced physiological stress, including reduced glucose, elevated lactate, and altered osmoregulatory function (lower urea and chloride, higher phosphorus). These patterns align with stress responses previously described in other shark species. Overall, the study provides a valuable baseline for future research into the physiology and conservation of Mediterranean blue sharks’ population.

## Introduction

Sharks play a crucial role in regulating marine ecosystems, influencing both coastal and oceanic community structures (Bradai *et al.*, 2023; Starostinetsky-Malonek *et al.*, 2023; Hammerschlag *et al.*, 2025). Although sharks are generally not the primary target of commercial longline fisheries, they are frequently caught as bycatch (*i.e.* the incidental capture of non-target species or small-sized individuals in non-selective fisheries; Gilman *et al.*, 2007; Skomal and Bernal, 2010; Biton-Porsmoguer and Lloret, 2018). These incidental catches can have a significant impact on their populations as the life history characteristics of sharks, including delayed maturity, prolonged gestation, pronounced maternal investment and reduced fecundity, make them particularly vulnerable to fishing pressure (Dulvy *et al.*, 2014; Bradai *et al.*, 2018; Prohaska *et al.*, 2021). As a result, elasmobranch populations are declining worldwide (Dulvy *et al.*, 2024; Worm *et al.*, 2024), but some species have a higher potential for population recovery if managed in a sustainable manner, such as the blue shark (*Prionace glauca*, Carcharhinidae), which has been observed to produce large litters (CITES Secretariat, 2024). The Mediterranean Sea is regarded as a region of significant concern for elasmobranchs, given its high proportion of threatened species and substantial decline in pelagic shark populations, attributable to elevated levels of human activity (e.g. fishing pressure, habitat degradation, chemical pollution; Micheli *et al.*, 2013; Gallo *et al.*, 2025) and to the vulnerability of shark species (Bradai *et al.*, 2012, 2018; Dulvy *et al.*, 2016; Abril *et al.*, 2020; Walls and Dulvy, 2021). In the Mediterranean, tuna and swordfish longline fisheries are responsible for a significant proportion of shark bycatch, particularly in the Alboran and Adriatic Seas (Bradai *et al.*, 2023; Carbonara *et al.*, 2023).

In pelagic longline fisheries, sharks are frequently caught alive (Hutchinson and Bigelow, 2019; Carbonara *et al.*, 2024), but capture and handling of sharks have been shown to induce acute stress that can disrupt their biochemistry and compromise post-release survival (Skomal, 2007; Skomal and Bernal, 2010; Gallagher *et al.*, 2014; Whitney *et al.*, 2021). Post-release mortality can be high due to injuries sustained during capture and handling, as well as the physiological costs of the stress response (Skomal, 2007; Skomal and Mandelman, 2012; Wosnick *et al.*, 2017; Bouyoucos *et al.*, 2018; Prohaska *et al.*, 2021). Consequently, there is an urgent need to deepen our understanding of stress physiology in commonly bycaught species and to improve our understanding of the stress responses exhibited by captured sharks. This improved knowledge can be useful for the refinement of fisheries management practices, as it will facilitate our ability to assess the physiological status of wild populations, help to identify the main causes of bycatch-induced mortality and assess current practices’ impact on individuals (Dapp *et al.*, 2016; Whitney *et al.*, 2021).

The stress response in fish has been extensively documented, with elasmobranchs exhibiting distinctive physiological adaptations such as corticosteroid mobilization (Anderson, 2012; Wosnick *et al.*, 2017). Essentially, a stressor triggers a primary neuroendocrine response, which in turn triggers the release of catecholamines (e.g. noradrenaline, epinephrine) and corticosteroids. These, in turn, activate secondary metabolic processes involving the mobilization and use of energy reserves, ultimately affecting individual fitness (tertiary response) (for details, see review in Skomal and Mandelman, 2012). The assessment of blood chemistry allows the evaluation of the range of variation of different parameters (Bouyoucos *et al.*, 2018; Prohaska *et al.*, 2021), including levels in individuals in bad condition (e.g. stressed, unhealthy individuals) and baseline values. However, obtaining authentic baseline values from wild shark individuals is challenging, as the capture process itself is a stressor. Instead, the categorization of physiological data can be based on the condition of captured individuals. Those that appear to be in good condition can provide ‘minimally stressed’ reference values (Prohaska *et al.*, 2021), while moribund individuals can help define upper stress thresholds (Moyes *et al.*, 2006; Wosnick *et al.*, 2017). Furthermore, it is important to assess the global physiological state of individuals, as some may be more susceptible to fishing mortality due to their pre-capture health status. This global assessment could facilitate the development of future proxies for predicting post-release survival and assessing the health status of sharks exposed to fisheries interactions. The stress response has been shown to vary between species (Mandelman and Skomal, 2009; Gallagher *et al.*, 2010; Hyatt *et al.*, 2012; Marshall *et al.*, 2012; Skomal and Mandelman, 2012), highlighting the need for targeted studies. Furthermore, it is imperative to investigate the intra-species variability of haematological and biochemical parameters. Indeed, baseline values and responses to stress may also vary within species, influenced by factors such as developmental stage, sex and season (Manire *et al.*, 2001; Hoffmayer *et al.*, 2012; Persky *et al.*, 2012; Valls *et al.*, 2016; Morón-Elorza *et al.*, 2022).

The blue shark is the most frequently caught species of shark by longline fisheries worldwide (up to 70–90% of the bycatch of sharks in pelagic longline fisheries; Oliver *et al.*, 2015). It represents a significant proportion of bycatch in the Mediterranean (Nakano and Stevens, 2008; Carbonara *et al.*, 2023, 2024), accounting for over 70% of elasmobranch longline catches (Bradai *et al.*, 2012; Carpentieri *et al.*, 2021), although it appears to be relatively more resilient to exploitation than other shark species due to its abundance, wide distribution, fecundity and its fast growth (Druon *et al.*, 2022 and references therein). The IUCN classifies the Mediterranean blue shark population as critically endangered (Dulvy *et al.*, 2016), and there are no recovery indicators (Biton-Porsmoguer and Lloret, 2018). The population is currently managed as a single stock, although recent evidence suggests the possibility of substructuring within the region (Leone *et al.*, 2024; Poisson *et al.*, 2024). Despite the fact that a number of studies have been conducted on the physiological characteristics of blue sharks (Emery, 1986; Wells *et al.*, 1986; Moyes *et al.*, 2006; Hight *et al.*, 2007; Marshall *et al.*, 2012; Harding *et al.*, 2022; Shea *et al.*, 2022), no such data are currently available for the Mediterranean population. Although it has poorly been directly investigated to date, intraspecific differentiation in physiological profiles, including interpopulation differentiation, could be expected, as demonstrated in other marine species (Karsten and Rice, 2004; Whiting *et al.*, 2007; Starostinetsky-Malonek *et al.*, 2023). This underlines the importance of conducting specific studies on this critically endangered population.

This study aimed to assess the extent of variation in a suite of physiological parameters associated with key biological functions in blue sharks incidentally caught in the Adriatic Sea longline fishery. These included markers of oxygen transport (e.g. haematocrit, haemoglobin), energy metabolism and stress response (e.g. adrenaline, lactate), protein metabolism and immune function (e.g. total protein, globulins), osmoregulation (e.g. urea, chloride), and lipid metabolism (e.g. cholesterol, non-esterified fatty acids [NEFA]). In the absence of baseline blood chemistry data, individuals were compared based on their post-capture condition to gain insights into physiological stress responses and to characterize the natural variability of blood and biochemical parameters over a multi-year dataset. Additionally, the influence of sex and ontogenetic stage (juvenile vs. adult) was analyzed across all measured parameters, offering critical and data-driven insights into the conservation and management of this critically endangered population in one of the most heavily impacted regions of the Mediterranean.

## Materials and Methods

The animals used in this study were obtained as incidental bycatch from authorized commercial fisheries. The sampling design and handling methods were reviewed and approved by the Committee on the Ethics of Animal Experiments of COISPA (Italian Ministry of Health 17/2022-UT).

### Sampling

**Figure 1:**
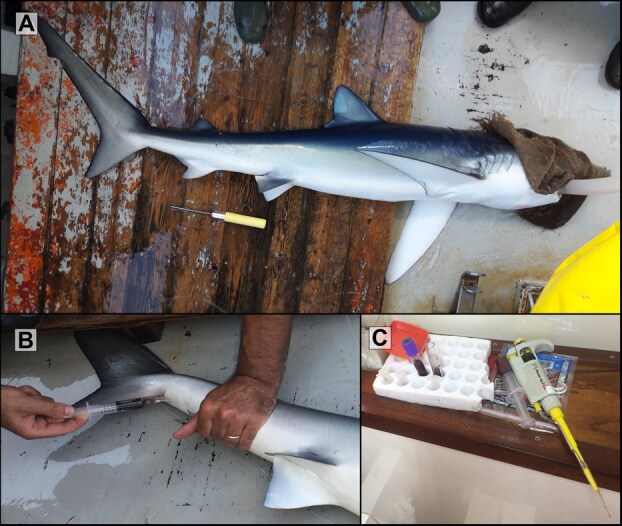
Steps after the capture of blue shark individuals. **A**) Individuals were blindfolded with a wet cloth and a tube was inserted into the mouth, into which water was pumped to maintain adequate gill oxygenation. **B**) Blood collection by caudal venipuncture. **C**) Blood kept in heparinized tubes

As part of the routine monitoring of the commercial pelagic longline fisheries and the tagging projects (SafeShark and MedByCatch) in the Adriatic Sea, blue shark individuals were blood sampled between 2020 and 2023. The longline (30–40 km long) was set at the start of the afternoon (3–4 pm) and was in place within approximately 3 hours. The longline haul back commenced at night and concluded in the morning, around 7–8 am. The hauling of the longline started with the retrieval of the final hooks. Consequently, the hooks were left at sea for a period ranging from 10 to 20 hours (the time between the last hook set at sea and the first hook recovered). The baits utilized in the present study comprised plastic squid filled with sardine (Fig. S1) and frozen mackerel (Scombridae) (Fig. S2) (Carbonara *et al.*, 2023). Once on board, the sharks were blindfolded with a wet cloth and a tube was inserted into their mouths, with water being pumped into them to maintain adequate gill oxygenation according to Poisson *et al.* (2012); Fig. 1A, Fig. S3). The blindfold has a slight sedative effect, which keeps the shark calm (Bruce and Bradford, 2012). The total length of each individual was measured. As soon as possible after the animal had landed on the ship’s deck, 2.5 ml of blood were sampled via caudal venipuncture using heparinized syringes (Fig. 1B-C). The interval between longline retrieval with hooked blue sharks and blood sampling was approximately two minutes. In the case of males for which sexual maturity could be assessed through an external examination, the pterygopods were examined in order to determine their stage (GFCM, 2016; AAVV, 2017). The categorization of individuals as juveniles or adults was determined by their size and when possible, also based on gonad maturity analysis (i.e. all males and in the case of deceased specimens for females). Globally, males and females were classified as juveniles if the total length was less than 150 and 180 cm, respectively, according to Megalofonou *et al.* (2009). Furthermore, for individuals with total lengths close to these limits captured dead, individuals were classified as juveniles if the maturity stage was 1 (immature) or 2 (immature, developing) and as adults for more advanced maturity stages (spawnable, actively spawning, pregnant, regressing and regenerating; AAVV, 2017). Following previous studies of Benoît *et al.* (2010) and Dapp *et al.* (2016), each individual was assigned a capture condition index, a numerical value indicating the health and well-being of the individual: 1 for individuals in good condition with vigorous body movements, 2 for sluggish individuals with weak body movements and responsiveness to touch and prodding, and 3 for moribund/dead individuals with no body movements and no responsiveness to touch and prodding.

### Sample processing

In total, 21 different parameters were assessed. Haematocrit was assessed using a microhaematocrit tube filled with blood directly from the syringe needle. Red blood cell count (RBCC) was performed in a Bürker counting chamber under a light microscope (Nikon 400E, Japan). To proceed, 5 μL of blood was diluted in 1 mL of Hendrix solution, and 18 independent counts were made by a single observer for each sample, with the results then averaged. Haemoglobin was measured using Drabkin’s reagent (H7379; Sigma, USA) following manufacturer’s recommendations and absorbances were read at 540 nm using an ELISA microplate reader (DAS srl, Rome, Italy). The remaining blood was transferred to a tube containing K3-EDTA (VACUMED, Torreglia, Italy) and plasma samples were collected after centrifugation and stored at −20°C until further analysis. Plasma cortisol was measured using a commercially available ELISA kit (E-70, InterMedical) according to the manufacturer’s instructions. Plasma glucose and lactate concentrations were measured using commercial enzymatic and colorimetric kits (4057 and 6751, respectively, Giesse Diagnostics) according to the manufacturer’s instructions. Immunoglobulin M levels were analyzed using an enzyme-linked immunosorbent assay kit (EA0025FI, BT LAB) according to the manufacturer’s protocol. IGF1 levels were assessed using two different enzyme-linked immunosorbent assay kits (E0016Bo and EA0022FI, BT LAB) according to the manufacturer’s guidelines. Adrenaline and noradrenaline were analyzed using competitive ELISA kits (EU2563 and EU2565, FineTest) according to the manufacturer’s instructions. Total protein, albumin, globulin, urea, cholesterol, triglycerides, aspartate aminotransferase, creatine kinase, phosphorus and chloride were measured in plasma on a Cobas Pure e303 analyser (Roche Diagnostics, Mannheim, Germany) using commercially available diagnostic kits from Roche Diagnostics (Table S1). The concentration of NEFA in shark plasma was measured by an enzymatic colorimetric method (Randox Laboratories Ltd, Crumlin, UK) using the Cobas Pure e303 analyser (Roche Diagnostics, Mannheim, Germany; Table S1).

### Statistical analyses

All statistical analyses were performed using the R software version 4.3.1 (R Core Team, 2023) at a 95% significance level. Data are expressed as mean ± sd (standard deviation), with fish individuals as the statistical unit. Creatine kinase values obtained for 24 samples were below the detection limit (*i.e.* <7) and were therefore arbitrarily set at 4 for statistical analyses. The majority of values obtained for cortisol and aspartate aminotransferase were below the methodological detection limits and these two parameters were therefore excluded from further statistical analyses. In addition, the high inter-assay coefficient of variation of IGF1 led to its exclusion from subsequent analyses.

In order to obtain a comprehensive global perspective of the influence of condition, sex and developmental stage on the physiological state of the individuals, a multivariate analysis was performed using a set of 18 variables, *i.e.* haemoglobin, haematocrit, RBCC, glucose, lactate, immunoglobulin M, total proteins, albumin, globulin, urea, cholesterol, triglycerides, NEFA, phosphorus, creatine kinase, chloride, adrenaline and noradrenaline. It should be noted that there were some missing values for the haemoglobin (*n* = 3), haematocrit (*n* = 2) and RBCC (*n* = 12), which corresponded to different individuals among variables. To address this, the missing values were estimated through the use of deterministic regression imputation (*mice* package; (Buuren and Buuren and Groothuis-Oudshoorn, 2011). Modelling imputation was also performed for adrenaline (*n* = 9) and noradrenaline (*n* = 5). All analyses were performed with the dataset containing only individuals without missing values and the full dataset with imputed values, and the overall conclusions did not change. Following data scaling, principal component analysis (PCA) was performed using the *FactoMineR* library (Le *et al.*, 2008)*.* The number of principal components to be retained for analysis was determined using the *nFactors* package (Raiche and Magis, 2022), analyzing results from the parallel analysis, the optimal coordinates and the acceleration factor methods. The PCA results were visualized using the *factoextra* library (Kassambara and Mundt, 2020). The principal component scores of the relevant axes were then extracted and Mann–Whitney-Wilcoxon/Student or Kruskal–Wallis/ANOVA tests were performed to assess the statistical difference between the groups. Finally, the variables that contributed most to each PCA component were identified using the *FactoMineR* library. The same method was used to assess the difference between the three condition indices, sex and life stage.

Subsequently, univariate analyses were conducted to investigate the differentiation between the condition groups in greater depth. For all linear models, graphical assessments were conducted to verify the assumptions of normality of residuals, linearity, absence of outliers, and homoscedasticity. Log-transformation was applied to glucose, total proteins, cholesterol, triglycerides, phosphorus, chloride and lactate data. To individually analyze the effect of condition on haematological and biochemical factors, linear models (*lme4* library; Bates *et al.*, 2014) or Kruskal–Wallis tests were used. Subsequent to the implementation of linear models, estimated marginal means post hoc tests were executed, accompanied by the Benjamini–Hochberg correction for multiple comparisons. Following the Kruskal–Wallis tests, the differentiation between condition groups was evaluated through the implementation of the Nemenyi’s non-parametric all-pairs comparison test (*PMCMRplus* library; Pohlert, 2024) with Benjamini–Hochberg correction for multiple comparisons.

## Results

A total of 63 blue shark individuals were blood sampled between 2020 and 2023 (Table 1).

**Table 1:** Characteristics of the sampled blue sharks

**Capture period**	**Number of males**	**Number of females**	**Life stage**	**Capture condition**	**Total length range (cm)**
August 2020	4	5	6 juveniles 3 adults	Condition 1: 3 Condition 2: 4 Condition 3: 2	139.5–174.7
September 2020	8	9	12 juveniles 5 adults	Condition 1: 8 Condition 2: 7 Condition 3: 2	83.5–195.5
October 2020	8	3	2 juveniles 9 adults	Condition 1: 6 Condition 2: 3 Condition 3: 2	132–219
August 2021	8	6	8 juveniles 6 adults	Condition 1: 8 Condition 2: 4 Condition 3: 2	138–223.7
November 2021	0	1	Adult	Condition 1	202.3
August 2022	0	4	3 juveniles 1 adult	Condition 1: 2 Condition 3: 2	126.6–181.2
September 2022	1	4	5 juveniles	Condition 1: 4 Condition 2: 1	94.1–165
August 2023	1	1	1 juvenile 1 adult	Condition 1: 2	126.6–180

**Figure 2:**
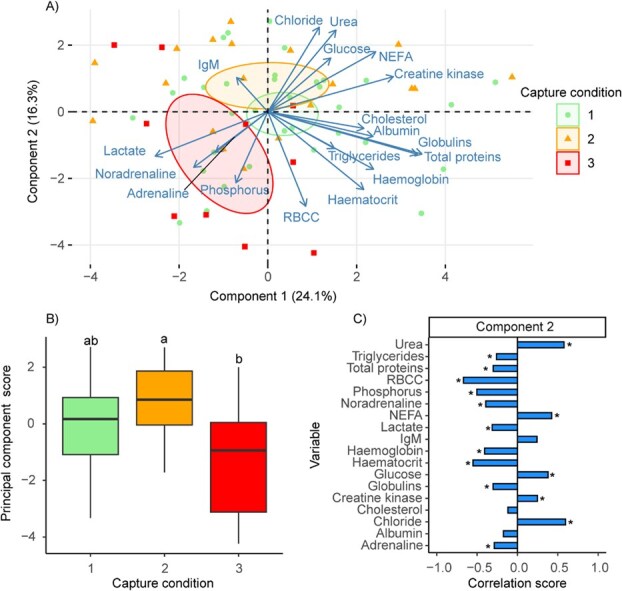
PCA. **A**) Visualization of individual fish positions on PCA components 1 and 2 as a function of condition index; confidence ellipses were drawn around condition groups with a confidence level of 0.95. **B**) PC scores for the second component as a function of condition; different letters in the box plots indicate a significant statistical difference between conditions (*P* < 0.05). **C**) Contribution of the eighteen variables to the second component of the PCA. Asterisks indicate variables that contribute significantly to the second component (*P* < 0.05). RBCC: red blood cell count; IgM: immunoglobulin M; NEFA: non-esterified fatty acids

**Figure 3:**
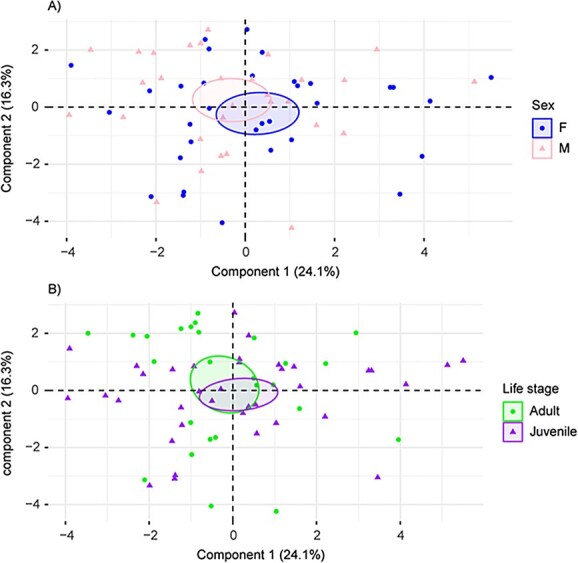
Visualization of the positioning of individual fish on the PCA components 1 and 2 as a function of: **A**) sex and **B**) life stage. Confidence ellipses were drawn around the condition groups with a confidence level of 0.95

The first two components of the PCA were retained and together accounted for 40.41% of the total variability of the data (Fig. 2A). The contribution of the different variables varied considerably within each component (Fig. 2A). Subsequent analysis of the principal component scores revealed that the first component did not show a statistically significant difference (*F* = 1.84, *P* = 0.17). However, a statistically significant difference between condition groups 2 and 3 was observed in the second component (*K* = 7.20, *P* = 0.03; Fig. 2B). Despite a slight separation in the confidence ellipses (Fig. 3), no significant differences could be found for any axis when comparing males and females (Axis 1: *t* = 1.10, *P* = 0.28; Axis 2: *W* = 407, *P* = 0.23) or when evaluating the life stage effect (Axis 1: *t* = −0.66, *P* = 0.51; Axis 2: *W* = 583, *P* = 0.15) (Fig. 3).

An examination of the variables contributing to the second PCA component (Fig. 2C) shows that RBCC was the most influential factor (15.23%), followed by chloride (12.21%), urea (11.41%), haematocrit (10.33%), phosphorus (8.61%), NEFA (6. 15%), haemoglobin (5.63%), noradrenaline (5.27%), glucose (4.93%), lactate (3.35%), globulins (3.08%), total proteins (3.06%), adrenaline (2.81%), triglycerides (2.31%) and creatine kinase (2.11%). The remaining variables did not show a significant association with the second component. On a global scale, individuals with a condition index of 3 had higher levels of lactate, adrenaline, noradrenaline, phosphorus, RBCC, haematocrit, haemoglobin, triglycerides, total proteins and globulins (Fig. 2A). Conversely, individuals in group condition 2 had higher levels of creatine kinase, chloride, urea, glucose and NEFA compared to condition 3 (Fig. 2A).

**Figure 4:**
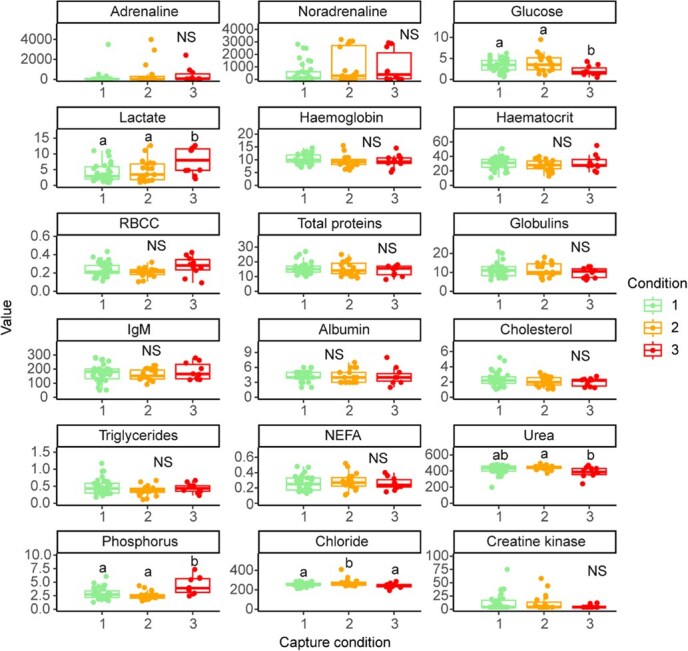
Box plots of the different physiological parameters according to condition groups. Dots represent the individual values. The central line of the boxplots indicates the median and the boxes indicate the quartiles, with the whiskers covering 95% of the values. Different letters on the box plots indicate a significant statistical difference between conditions (*P* < 0.05). NS: no statistical difference. Units: adrenaline and noradrenaline in pg·ml^−1^, glucose and lactate in mmol·l^−1^, haemoglobin in g·dl^−1^, haematocrit in %, RBCC in × 10^6^·μl^−1^, total proteins, albumin and globulin in g·l^−1^, IgM (immunoglobulin M) in μg·ml^−1^, cholesterol in mmol·l^−1^, triglycerides in mmol·l^−1^, NEFA in mEq·l^−1^, urea, phosphorus and chloride in mmol·l^−1^, and creatine kinase in U·L^−1^

When evaluating the effect of condition on each parameter, statistical differences between conditions were found for glucose (*F* = 4.91, *P* = 0.01), lactate (*F* = 3.80, *P* = 0.03), urea (*K* = 6.99, *P* = 0.03), phosphorus (*F* = 10.70, *P* < 0.001) and chloride (*F* = 4.66, *P* = 0.01) (Fig. 4). Moribund/dead individuals were significantly different from the other groups for glucose, lactate and phosphorus while the group condition 2 is significantly different from the two other ones in chloride and urea was significantly lower in moribund/dead individuals compared to condition 2 (Fig. 4). There were no statistically significant differences between the condition groups for adrenaline (*K* = 3.45, *P* = 0.18), noradrenaline (*K* = 1.24, *P* = 0.54), haemoglobin (*F* = 1.30, *P* = 0.28), haematocrit (*F* = 0.68, *P* = 0.51), erythrocyte count (*F* = 2.76, *P* = 0.07), total protein (*F* = 0. 53, *P* = 0.59), globulin (*K* = 1.01, *P* = 0.60), immunoglobulin M (*F* = 0.60, *P* = 0.55), albumin (*K* = 0.92, *P* = 0.63), cholesterol (*F* = 1.28, *P* = 0.29), triglycerides (*F* = 1.96, *P* = 0.15), NEFA (*F* = 0.35, *P* = 0.71) and creatine kinase (*K* = 1.71, *P* = 0.42) (Fig. 4).

## Discussion

This study presents, for the first time, a detailed assessment of the range of variation of haematological and biochemical parameters in blue sharks caught in the Mediterranean Sea, based on the most extensive physiological dataset available for this species. Establishing baseline values for wild-caught individuals is inherently challenging, as the capture and handling process induces stress and triggers physiological changes (Wosnick *et al.*, 2017). Recent work has proposed that post-mortem values could serve as a reference point, with individuals showing values closer to this group potentially experiencing higher stress levels (Wosnick *et al.*, 2017). However, relying solely on stress markers is not sufficient to determine the pre-capture condition of individuals, and a broader set of physiological indicators must) be considered. In this study, we observed clear differences in physiological state between the defined condition groups.

Most of the haematological and biochemical values recorded fell within the ranges previously reported for shark species (Mandelman and Skomal, 2009; Hyatt *et al.*, 2012), including shark species from the same geographic zone (Falco *et al.*, 2023; Starostinetsky-Malonek *et al.*, 2023), although inter-species variability can be observed, and other blue shark populations from other geographic areas (Hight *et al.*, 2007; Marshall *et al.*, 2012; Moyes *et al.*, 2006; Wells *et al.*, 1986; Table S2). IGF-1 and aspartate aminotransferase were excluded from the analysis due to values below detection limits or high inter-assay variability. Growth hormone, a likely key regulator of elasmobranch growth, has been isolated from the pituitary of blue sharks; however, the specific role of IGF-1 in this group remains poorly understood (Gelsleichter and Evans, 2012). Reported differences in aspartate aminotransferase levels in the literature (Moyes *et al.*, 2006) may reflect methodological inconsistencies or natural variability among individuals or populations.

While being hooked and handled, individual sharks may be exposed to hypoxia or even anoxia, causing oxidative stress and leading to a cascade of responses (Renshaw *et al.*, 2012). In sharks, catecholamines have been shown to rise significantly immediately after the perception of the stressor, peaking in the minutes following the end of the stress event (Skomal and Bernal, 2010). With the exception of a small number of individuals displaying elevated levels of adrenaline and noradrenaline, the overall levels reported here were low. These values can be strongly influenced by the length of time spent on the hook in the longline. Although not significant in univariate analyses, the trend in the PCA seen for the increase in catecholamines in moribund/dead individuals compared to those in better condition is consistent with that seen in other blue shark populations (Hight *et al.*, 2007). This trend has also been observed in other species, including the spotted dogfish (*Scyliorhinus canicula*) (Murray *et al.*, 2015) and the shortfin mako (*Isurus oxyrinchus*) (Hight *et al.*, 2007). As adrenaline is light-sensitive, despite keeping all plasma samples in the dark, we cannot rule out photo-degradation and loss of analyte occurring during the sampling process, which could also partly explain why most samples display very low values. Further investigation is required to eliminate this potential source of bias. With regard to corticosteroids, elasmobranchs appear to have a functional hypothalamic–pituitary-interrenal axis, as stress exposure has been shown to be regulated by the expression of the pituitary factor adrenocorticotropic hormone (Skomal and Bernal, 2010; Gelsleichter and Evans, 2012). Our results confirm what has been shown previously for various shark species, that unlike teleosts, cortisol is not involved in the hypothalamic–pituitary-interrenal axis, as most of the samples analyzed were below the detection limit. Instead, 1α-hydroxycorticosterone is thought to be the corticosteroid involved in the response to stress (Pankhurst, 2011; Anderson, 2012; Gelsleichter and Evans, 2012; Wosnick *et al.*, 2017), but due to the difficulty in measuring this hormone (*i.e.* lack of available commercial ELISA kits), an alternative might be to measure its precursor, corticosterone, although the latter is also involved in reproductive cycles and the interpretation of variations could be biased, and its involvement in the stress response remains controversial (Manire *et al.*, 2007; Wosnick *et al.*, 2017).

Parameters involved in the secondary response to stress are more traditionally used as stress markers (Skomal and Bernal, 2010). We here reported a significant difference seen for five parameters, namely glucose, lactate, urea, phosphorus and chloride. Blood glucose levels between 3–20 mmol.L^−1^ were reported in literature after capture (Table S2), which is consistent with what was observed in this study for blue shark individuals with condition indices of 1 or 2. Glucose was seen to be lower in the group of moribund/dead sharks, in agreement with what was previously seen in other shark species, such as tiger shark (*Galeocerdo cuvier*; Wosnick *et al.*, 2017), bonnethead shark (*Sphyrna tiburo*) and bull shark (*Carcharhinus leucas*) (Manire *et al.*, 2001). Lipid-derived ketone bodies and amino acids constitute the major energetic fuelling source rather than glucose in sharks (Ballantyne, 2015). Instead, glucose is used as metabolic fuel for homeostatic balance rather than for aerobic metabolism (Wosnick *et al.*, 2017), which would be congruent with a decrease in moribund/dead individuals associated with a change in osmoregulation compounds (see urea and phosphorus further in the discussion). The plasma lactate concentration was about 1.8 times higher in moribund/dead sharks compared to the two other condition groups. A lactate increase following capture could be expected due to increased energetic demands and the mobilization of the anaerobic metabolism, as evidenced by patterns observed in tiger sharks (Wosnick *et al.*, 2017), spiny dogfish (*Squalus acanthias*; Mandelman and Farrington, 2007), white shark (*Carcharodon carcharias*; Tate *et al.*, 2019), bonnethead, lemon shark (*Negaprion brevirostris*) and bull shark (Hyatt *et al.*, 2012). Indeed, exhaustive exercise linked to the struggling on the longline during capture triggers anaerobic glycolysis (Skomal and Bernal, 2010; Hyatt *et al.*, 2012; Marshall *et al.*, 2012; Skomal and Mandelman, 2012). Values reported for lactate in moribund/dead individuals are however not very high compared to what was observed in blue shark after a stress event (Table S2; e.g. 4.7 time increase in Moyes *et al.*, 2006). Lactate is a very commonly used parameter for studying stress, but it is largely driven by shifts between aerobic/anaerobic metabolisms and therefore greatly influence by the duration that the shark spent in the net and the flight time during capture (Skomal, 2007; Mandelman and Skomal, 2009). All animals were captured and handled in the same way but it was not possible to assess the specific capture duration of each individual sampled, potentially contributing to the inter-individual variability observed. In addition, the lactate rise seen in blue sharks in response to stress is lower compared to other species, especially lamnid sharks (Marshall *et al.*, 2012). The lactate response appears to be highly species-specific (Marshall *et al.*, 2012 and references within) and carcharhinids (e.g. blue shark) were reported to exhibit a lower stress response than lamnid sharks, potentially due to lower anaerobic capacities for burst swimming (Skomal and Bernal, 2010; Marshall *et al.*, 2012). Regarding osmotic homeostasis, the patterns of plasma ions in response to stress appears to be species-specific (Mandelman and Farrington, 2007; Moyes *et al.*, 2006; Tate *et al.*, 2019; Wosnick *et al.*, 2017; see Table 2 in Wosnick *et al.*, 2017). The observed decrease in plasma urea levels in moribund/dead individuals is consistent with previous studies on other species (Mandelman and Farrington, 2007; Marshall *et al.*, 2012; Wosnick *et al.*, 2017), suggesting an inability to maintain the urea balance, potentially caused by the breakdown of renal urea reabsorption, loss of retention capacity, and membrane rupture in moribund/dead individuals (Wosnick *et al.*, 2017). Elevated phosphorus levels in moribund/dead sharks are also congruent with previous studies in severely stressed individuals, with*.* Significantly elevated values (i.e. 33 times higher than those observed in living individuals) recorded in moribund tiger sharks (Wosnick *et al.*, 2017). The increase of ions may result from the intracellular acidosis that reflects a myotomal or myocardial cell damage or potentially the loss of osmoregulatory capacities during the stress event (Moyes *et al.*, 2006; Marshall *et al.*, 2012). More specifically, hyperphosphatemia has been demonstrated to be highly detrimental to individuals, as it results in artery blockage through the formation of phosphate crystals. High phosphorus levels in plasma have been associated with cellular membrane disintegration, failure of the renal system, and gill collapse (Wosnick *et al.*, 2017). Although the increase observed in this study in moribund/dead individuals is modest (1.7 times increase compared to other condition groups), it still suggests that hyperphosphatemia indicates late-stage systemic failure or irreversible damages, as previously suggested in tiger sharks (Wosnick *et al.*, 2017). Finally, chloride, which plays a crucial role in the electroneutrality of the bloodstream (Skomal and Mandelman, 2012), was significantly lower in moribund/dead sharks compared to condition 2 individuals, contrarily to the absence of difference observed in Moyes *et al.* (2006). A strong inter-species variability was seen for chloride response to stress (Mandelman and Farrington, 2007; Skomal and Mandelman, 2012; Wosnick *et al.*, 2017; Tate *et al.*, 2019) and the use of chloride as a reliable stress indicator remains to be assessed. Another ion that would be interesting to investigate is the ion potassium, which provides an intracellular acidosis marker and has been previously used as a stress marker in sharks (Wells *et al.*, 1986; Manire *et al.*, 2001; Moyes *et al.*, 2006; Brooks *et al.*, 2012; Marshall *et al.*, 2012; Wosnick *et al.*, 2017).

Out of 63 individuals, 10 sharks belonged to the condition 3 group, indicating an overall good resistance to stress induced by bycatch, which is also supported by the low variation range of physiological parameters comparatively to other shark species. The low sensibility of blue sharks to bycatch was previously suggested (Moyes *et al.*, 2006; Shea *et al.*, 2022), with individuals captured by longlines displaying a substantially lower mortality rate compared to other species (Skomal and Bernal, 2010). This pattern could be confirmed by assessing other complementary stress proxies, such as heat shock proteins, pH or haemoxygenase (Moyes *et al.*, 2006; Marshall *et al.*, 2012; Renshaw *et al.*, 2012; Wosnick *et al.*, 2017). The five parameters differentiating condition groups could be used to identify individuals presenting a disruption in the homeostatic level. However, they cannot be used as a survival predictive tool as the presence of a stress response, associated with changes in acid–base balance and electrolyte dysregulation, is not necessarily highly detrimental to individuals. For instance, Mandelman and Farrington (2007) showed that the spiny dogfish shark presents a low mortality rate after capture, handling and transport despite the fact that individuals appeared as stressed (e.g. variations in lactate, pH, urea, total protein, haematocrit and electrolytes levels). A future study is required to evaluate the direct link between physiological parameters and post-release survival of blue sharks by sampling blood after capture and following for instance released individuals using tags to assess post-release survival. A better knowledge of factors leading to mortality can lead to the identification of threshold for stress to predicting post-release fate (Molina and Cooke, 2012). Moreover, in this context, enhanced awareness and training among fishers could be important to potentially further contribute to the reduction of post-capture mortality.

Other parameters were not reported to be significant in univariate analyses, although they were significant variables in the PCA analysis. Regarding haematological parameters linked to oxygen transport, the lack of haemoconcentration is congruent with what was previously observed in blue shark (Moyes *et al.*, 2006; but see a haematocrit decrease in Hight *et al.*, 2007) and in other species (Hoffmayer and Parsons, 2001; Manire *et al.*, 2001; Hoffmayer *et al.*, 2012; Fuller *et al.*, 2020), although PCA results show that further analyses are required. Delivery of oxygen to tissues is a key process determining the efficiency of aerobic processes. Due to the lack of adrenergic splenic contraction, that explains in teleosts a sharp rise of haematocrit in response to exercise, an absence of variation in haematocrit in response to hypoxia is usually expected in elasmobranch species (Ballantyne, 2015) (but see counter-examples in Frick *et al.*, 2012 and Mandelman and Farrington, 2007). Haematological parameters were higher than those previously reported in blue sharks (Marshall *et al.*, 2012; Moyes *et al.*, 2006; Wells *et al.*, 1986; but see high values reported in Hight *et al.*, 2007), but this could be due to intraspecific variability and compensatory mechanisms to acute stress (Skomal and Mandelman, 2012). Creatine kinase did not indicate muscle degradation, in accordance with the findings previously reported in the blue shark (Moyes *et al.*, 2006). The trend of higher creatine kinase activity in the condition 2 group compared to moribund/dead individuals seen in the PCA could be explained by the fact that moribund/dead sharks often present metabolic dysfunction, reducing the ability of damaged cells to release the enzyme into the bloodstream (Baldissera and Baldisserotto, 2023). Finally, regarding immune parameters, immunoglobulin M was the first to be identified as part of the humoral immunity of elasmobranchs (Luer *et al.*, 2004) and represents a substantial proportion of total proteins (Gargano *et al.*, 2025). In the present study, no distinction was identified between the condition groups for immunoglobulin M, total globulins or total proteins in univariate analyses. This finding suggests that health status did not appear to influence the classification across condition index groups. A lack of total protein variation in response to stress was previously shown in tiger shark (Wosnick *et al.*, 2017). However, the PCA results for moribund/dead blue sharks reveals a trend of lower immunoglobulin M, yet higher total protein and globulin. This suggests the necessity for further consideration of immune parameters, including other globulins such as gamma and beta globulin, which may offer a partial explanation for the variations observed in the PCA. A significant variability was also observed for the three immune parameters among the individuals, which may indicate underlying health status differences prior to capture, although this variability was not a determining factor in the post-capture condition. Overall, taking into account additional health indicators, such as innate immunity parameters (e.g. phagocytic activity, complement proteins; Luer *et al.*, 2004), freshness index or histopathological indicators (Wosnick *et al.*, 2020) could better unveil the immune status of wild blue sharks.

Interestingly, it was previously reported that elasmobranchs lack the NEFA binding protein albumin, which can be explained by the need to improve the glomerular filtration rate or the potential disruption of urea by the albumin hydrophobic site (Ballantyne *et al.*, 1993; Ballantyne, 2015). Most NEFA are thought to be transported by plasma lipoproteins (Ballantyne, 2015). However, we detected albumin (4.2 ± 1.2 g·L^−1^), which is consistent with the levels reported by Moyes *et al.* (2006). This is also congruent with levels found in NEFA (between 110 and 520 μmol·L^−1^; similarly to Atlantic blue shark values in Ballantyne *et al.*, 1993), which are elevated compared to those observed in other elasmobranchs (Speers-Roesch and Treberg, 2010), in contrast to the expected outcomes in the absence of albumin. It was previously suggested that elasmobranchs possess albumin-like proteins, which belong to glycoproteins (Andreeva, 2010), but their role remains to be investigated.

Finally, it is essential to understand the intrinsic (e.g. sex, developmental stage) and extrinsic factors (e.g. prey availability, environmental factors, chemical pollution, parasitic pressure) that influence the energetic state and condition of sharks. This has been well studied in teleosts (Ahmed *et al.*, 2020), but has received less attention in elasmobranchs. In this paper, we tested the potential effect of sex and life stage (juveniles vs. adults), focusing our attention on intrinsic characteristics. The present study found no difference between juveniles and adults, which is different from what has been previously observed in sand tiger sharks (*Carcharias taurus*; Hoopes *et al.*, 2022) and in blue sharks off the coast of Massachusetts, for which an inverse relationship between total length and glucose has been demonstrated (Shea *et al.*, 2022). The absence of difference in health or susceptibility to bycatch-induced stress between juveniles and adults is, however, consistent with previous studies on nurse sharks (*Ginglymostoma cirratum*; Moorhead *et al.*, 2021; Shinder *et al.*, 2022). No sex effect was shown for the blue shark caught in the Mediterranean Sea, while a sex effect has been reported for other species, such as the blacktip reef shark (*Carcharhinus melanopterus*; Mills *et al.*, 2024) or the nursehound shark (*Scyliorhinus Stellaris*; Morón-Elorza *et al.*, 2022). There is evidence that energetic and immune parameters vary by sex during the breeding season in other species (Sueiro *et al.*, 2019; Morón-Elorza *et al.*, 2022; Mills *et al.*, 2024). Given that the sharks under study here were captured outside the breeding season, it is possible that a sex effect may also be observed at other times of the year. Energetic parameters are also expected to vary with feeding frequency and during energetically demanding periods (Hoffmayer *et al.*, 2012; Gallagher *et al.*, 2017). Furthermore, the metabolism of sharks is influenced by various environmental factors, including temperature, salinity, dissolved oxygen levels and light intensity (Hoffmayer and Parsons, 2001; Carlson *et al.*, 2004; Frick *et al.*, 2010), or chemical pollution (Alves *et al.*, 2022). The sharks studied herein were caught at different periods (see Table 1). This may partly explain the inter-individual variability. In future research, the integration of environmental data with physiological data sets could facilitate a more comprehensive understanding of the observed inter-individual variability (see Starostinetsky-Malonek *et al.*, 2023).

## Conclusion

This study establishes the first physiological dataset for the Mediterranean blue shark population, a critically endangered species, providing a crucial foundation for future research and conservation efforts. The finding that a significant proportion of individuals exhibited low physiological disruption reinforces the assumption that blue sharks may possess a relatively high resilience to capture-related stress, at least in the short term. These results could still be useful in assessing the health status of blue sharks in the Mediterranean over the long term. They could also be used to identify and promote low-impact fishing and handling practices for blue sharks caught as bycatch in the Mediterranean commercial fisheries. In addition, they could be useful to assess the potential impact of other anthropogenic activities or environmental changes. In order to capture the full range of variability, particularly during key energetic moments such as reproduction and post-spawning, it is important to expand the dataset with additional individuals across other time periods. This will allow us to assess whether vulnerability to bycatch is higher during certain life stages at certain times. In order to ensure effective conservation planning, it is also crucial to gain a better understanding of how physiological variables relate to both intrinsic and extrinsic drivers and future studies should explore correlations with environmental conditions across a large timespan.

## Supplementary Material

Toomey_et_al_R1_Supplementary_materials_coaf067

## Data Availability

Datasets are available from the corresponding author on reasonable request.
