# Supplementary material for: Unveiling the biochemical and haematological profile of blue shark (Prionace glauca) in the Mediterranean after bycatch
Source: Conserv Physiol. 2025 Sep 20;13(1):coaf067. doi: 10.1093/conphys/coaf067 (PMC12449297; doi:10.1093/conphys/coaf067)

Unveiling the biochemical and haematological profile of blue shark (*Prionace glauca*) in the Mediterranean after bycatch

Lola Toomey, Andrea Bellodi, Massimiliano Bottaro, Antonella Consiglio, Eleonora Fiocchi, Margherita Soncin, Valentina Bertazzo, Maria Cristina Follesa, Amedeo Manfrin, Simone Niedermüller, Giulia Prato, Pierluigi Carbonara

SUPPLEMENTARY DATA

Table S1. Characteristics of methods used to measure a set of physiological parameters.

Parameter	Analytical method	Analytical sensitivity	Linearity interval
**Total protein**	Biuret	2 $g.L^{-1}$	2-120 $g.L^{-1}$
**Albumin**	Bromine cresol green (BCG)	2 $g.L^{-1}$	2-60 $g.L^{-1}$
**Globulin**	Calculation: difference between Total Protein and Albumin	-	-
**Urea**	UV kinetic Urease, glutamate dehydrogenase	0.5 $mmol.L^{-1}$	0.5-40 $mmol.L^{-1}$
**Cholesterol**	Colorimetric enzymatic CHOD-PAP	0.1 $mmol.L^{-1}$	0.1-20.7 $mmol.L^{-1}$
**Triglycerides**	Colorimetric enzymatic GPO-PAP	0.1 $mmol.L^{-1}$	0.1-10.0 $mmol.L^{-1}$
**Non-esterified fatty acids**	Colorimetric AcylCoA synthetase, AcylCoA ox., TOOS	0.072 $mmol.L^{-1}$	0.1-0.9 $mmol.L^{-1}$
**Aspartate aminotransferase**	IFCC with activation by pyridoxal phosphate (P5'P)	5 $U.L^{-1}$	5-700 $U.L^{-1}$
**Creatine kinase**	IFCC	7 $U.L^{-1}$	7-2000 $U.L^{-1}$
**Phosphorus**	UV complexometry Phosphomolybdate without reduction	0.10 $mmol.L^{-1}$	0.10-6.46 $mmol.L^{-1}$
**Chloride**	Indirect potentiometry	60 $mmol.L^{-1}$	60-140 $mmol.L^{-1}$

Table S2. Physiological studies performed on blue shark - *AST: Aspartate aminotransferase / CK: Creatine Kinase / RBCC: Red Blood Cell Count / NEFA: Non-Esterified Fatty Acids / IgM: Immunoglobulin M / - :* Information not available.

Study	Location	N	Animal state / Blood collection	Total length/ weight	Parameter	Range of variation (or if not available mean ± sd)
Shea et al., 2022	North Atlantic	20	Good condition – Blood sampled within 1-32.5 min after the shark was hooked	168 - 314 cm	Glucose	5.3 - 7.5 mmol.L^{-1}
					Lactate	0.5- 4.9 mmol.L^{-1}
					Phosphate	4.2 - 11.1 mg.dL^{-1}
Marshall et al., 2012	North Atlantic - Pacific	40	-	-	Chloride	265 ± 18 mmol.L^{-1}
					Glucose	6.0 ± 1 mmol.L^{-1}
					Lactate	4.8 ± 4 mmol.L^{-1}
					Haematocrit	18.7 ± 4.5 %
Harding et al., 2022	North Atlantic	14	-	159 - 237 cm	Lactate upon capture	1.2 - 9 mmol.L^{-1}
					Lactate prior to release	2.9 - 9 mmol.L^{-1}
					Glucose upon capture	3.1 - 19.7 mmol.L^{-1}
					Glucose prior to release	2.9 - 22.7 mmol.L^{-1}
Emery, 1986	North Atlantic	18-19	-		Haemoglobin	1.9 - 4.7 mmol.L^{-1}
					Haematocrit	9.4 - 22.5 %
Moyes et al., 2006	Pacific	16-20	7-9 moribund, 9-11 individuals in good condition that were tagged and survived after release		Lactate from moribund	27.72 ± 4.07 mmol.L^{-1} (se)
					Lactate from survivors	5.80 ± 2.96 mmol.L^{-1} (se)
					Glucose from moribund	4.16 ± 0.69 mmol.L^{-1} (se)
					Glucose from survivors	4.75 ± 0.40 mmol.L^{-1} (se)
					Chloride from moribund	236 ± 5 mmol.L^{-1} (se)
					Chloride from survivors	240 ± 4 mmol.L^{-1} (se)
					Urea from moribund	352 ± 11 mmol.L^{-1} (se)
					Urea from survivors	357 ± 4 mmol.L^{-1} (se)
					AST from moribund	26.9 ± 11.2 U.L^{-1} (se)
					AST from survivors	38.3 ± 22.3 U.L^{-1} (se)
					CK from moribund	537 ± 490 U.L^{-1} (se)
					CK from survivors	3468 ± 2282 U.L^{-1} (se)
					Protein from moribund	11.4 ± 0.9 g.L^{-1} (se)
					Protein from survivors	14.6 ± 0.6 g.L^{-1} (se)
					Albumin from moribund	3 ± 0.22 g.L^{-1} (se)

					Albumin from survivors	2.60 ± 0.24 g.L^{-1} (se)
					Haematocrit from moribund	16.1 ± 1.5 % (se)
					Haematocrit from survivors	19.4 ± 2.4 % (se)
Hight et al., 2007	Pacific	2-60 depending on the parameter	Blood collected within 2-15 min after capture	123 ± 3 cm	Lactate after tagging<br>Lactate from dead/moribund<br>Haematocrit after capture<br>Haematocrit after tagging<br>Haematocrit from dead/moribund<br>Adrenaline after capture<br>Adrenaline after tagging<br>Adrenaline from dead/moribund<br>Noradrenaline after capture<br>Noradrenaline after tagging<br>Noradrenaline from dead/moribund	1-26 mmol.L^{-1}<br>1-40 mmol.L^{-1}<br>19-26 %<br>4 - 31 %<br>4 - 20 %<br>432 - 1349 pg.mL^{-1}<br>558 - 75625 pg.mL^{-1}<br>4305 - 131988 pg.mL^{-1}<br>678 - 2199 pg.mL^{-1}<br>463 - 51715 pg.mL^{-1}<br>5232 - 97418 pg.mL^{-1}
Wells et al., 1986	New-Zealand - Pacific	2	-	104 kg	Chloride<br>Phosphate<br>Glucose<br>Lactate<br>Cholesterol<br>Urea<br>Whole blood haemoglobin<br>Haematocrit<br>RBCC<br>AST<br>CK<br>Albumin<br>Total protein	291 mmol.L^{-1}<br>18 mmol.L^{-1}4.5 mmol.L^{-1}<br>9 mmol.L^{-1}<br>1 mmol.L^{-1}<br>294 mmol.L^{-1}<br>29 g.L^{-1}<br>11 %<br>0.23 x 10^{6} .µL^{-1}<br>44<br>117<br>4 g.L-1<br>8 g.L-1
Current study	Adriatic Sea	63	34 sharks of condition 1, 19 sharks of condition 2, 10 sharks of condition 3		Haemoglobin<br>Haematocrit<br>Red blood cell count<br>Glucose<br>Lactate<br>IgM<br>Total proteins<br>Albumin	5.1-15-5 g/dL<br>10.7-55.1 %<br>0.09-0.43 x 10^{6} /µL<br>0.45-9.5 mmol.L-1<br>0.91-12.68 mmol.L-1<br>50.62-282-61 µg.mL-1<br>8-27 g.L-1<br>2-8 g.L-1

	Globulin	6-21 g.L-1
	Urea	199-494 mmol.L-1
	Cholesterol	1.05-5.23 mmol.L-1
	Triglycerides	0.11-1.17 mmol.L-1
	NEFA	0.11-0.52 mEq.L-1
	Phosphorus	1.30-7.36 mmol.L-1
	Chloride	194-410 mmol.L-1
	Adrenaline	4.24-4000 pg.mL-1
	Noradrenaline	0-3194.91 pg.mL-1
	Creatine kinase	<7 – 75 U.L-1

Supplementary figures

Figure S1. Bait used. Plastic squid filled with sardines.

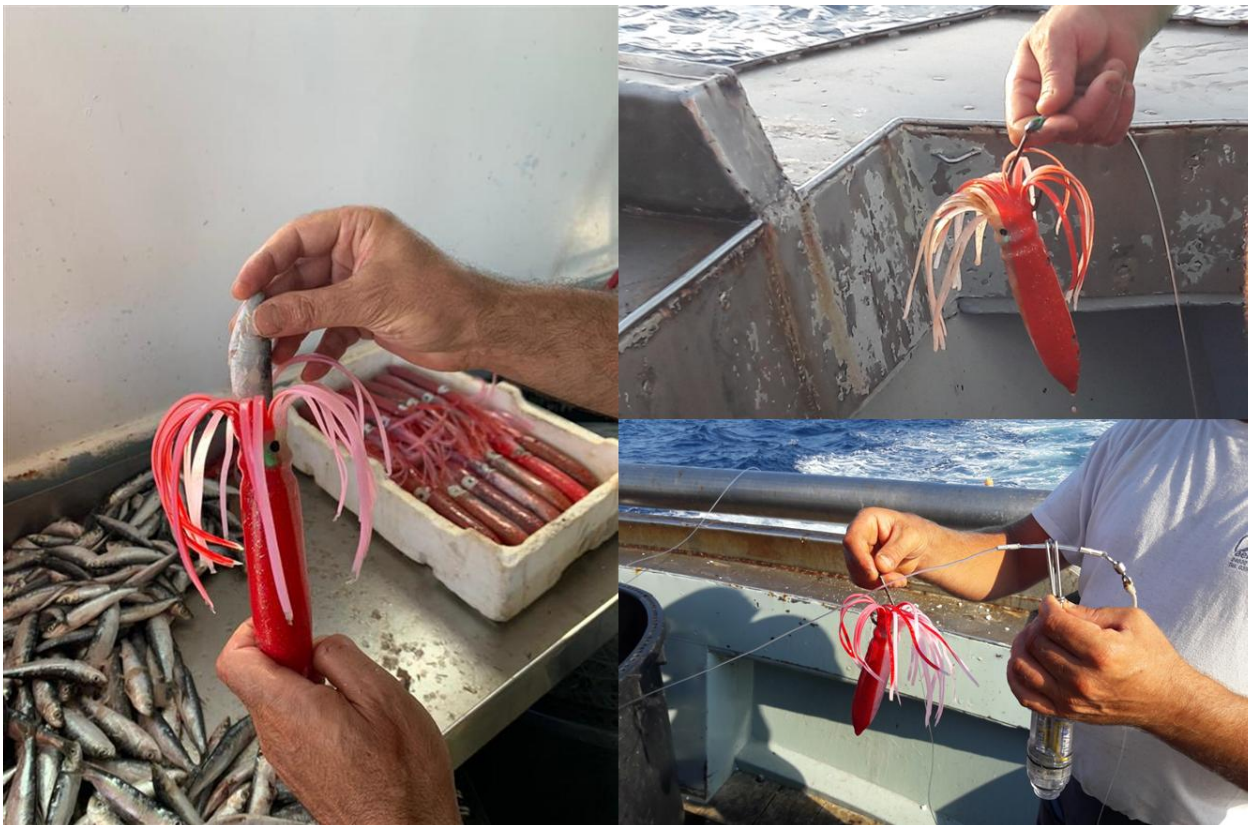

Figure S2. Bait used. Frozen mackerel

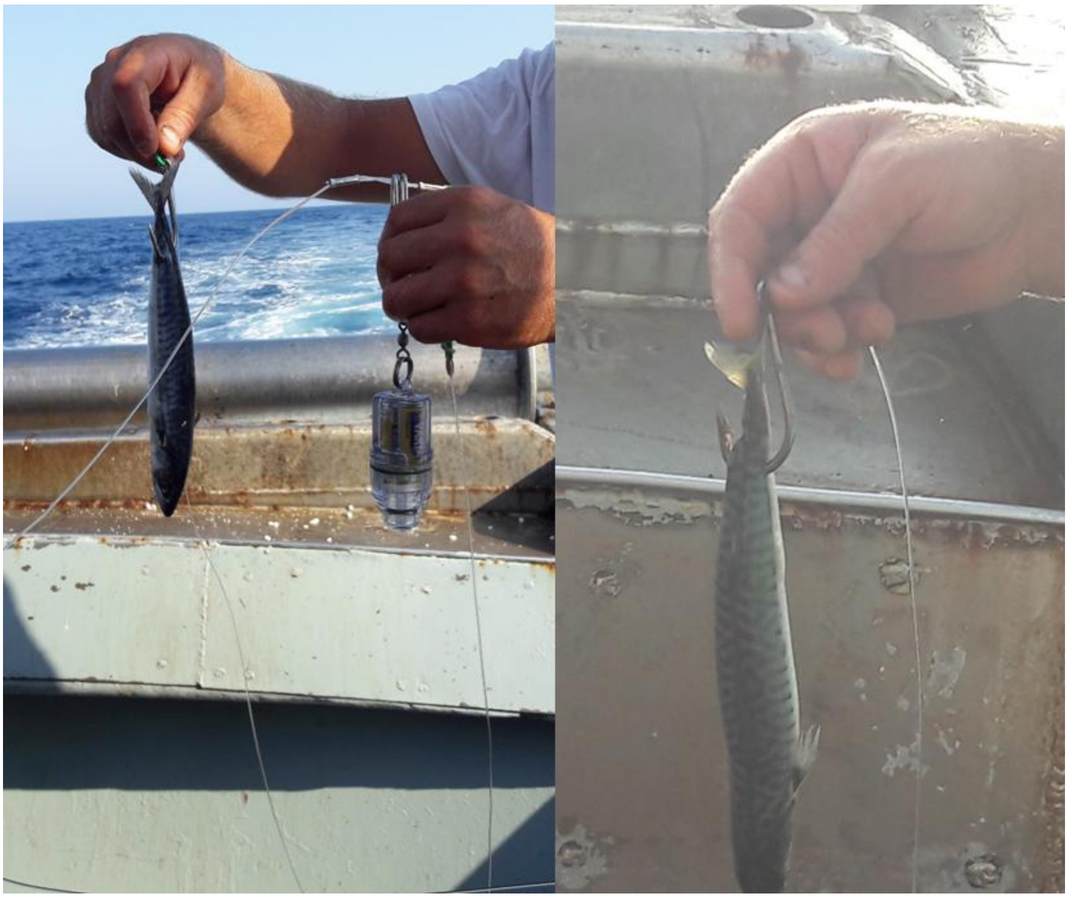

Figure S3. By-caught blue sharks.

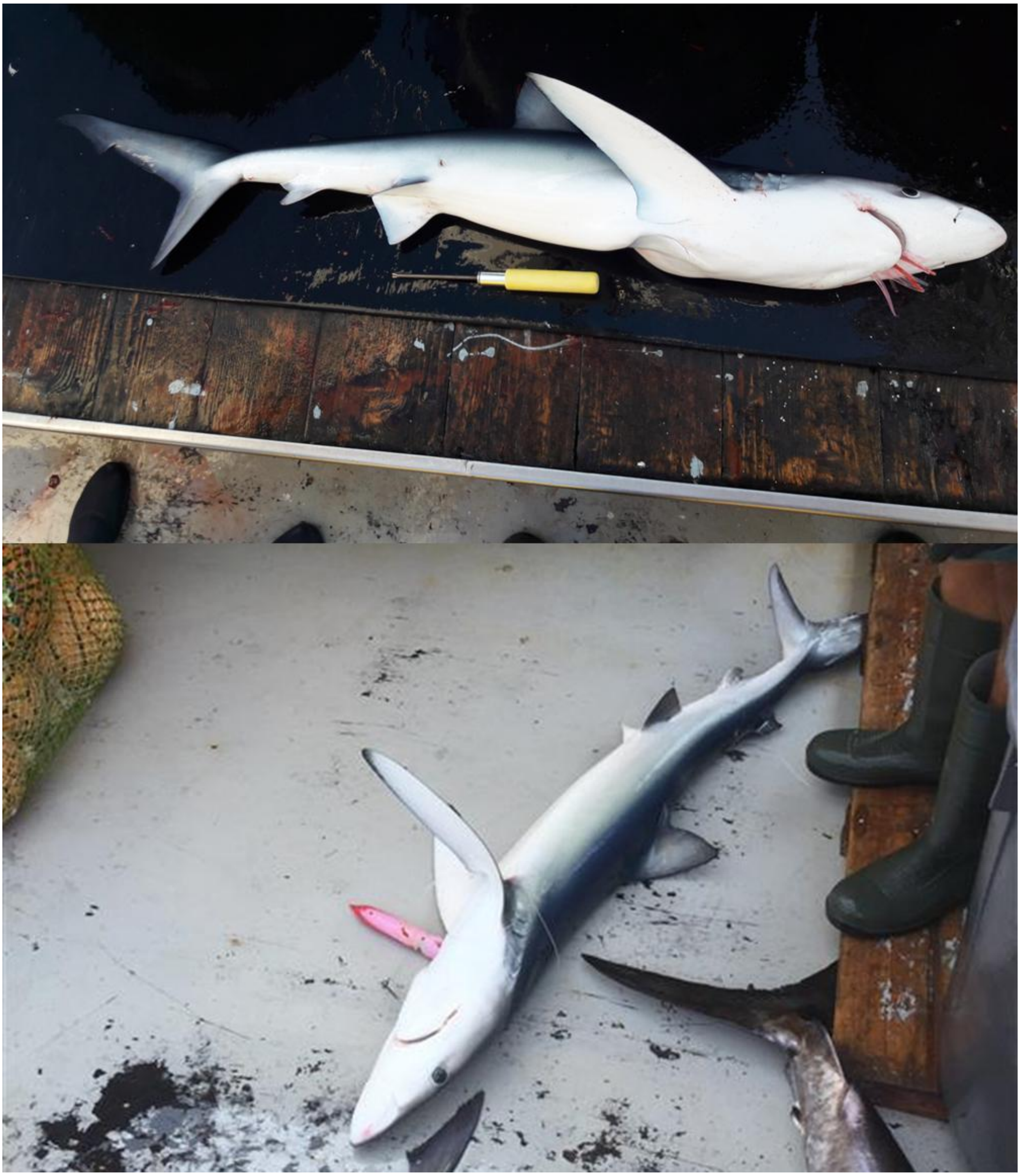

Supplement: Toomey_et_al_R1_Supplementary_materials_coaf067 [file toomey_et_al_r1_supplementary_materials_coaf067.pdf]
